# Comparative Analysis of Fluorescence Emission in Myricetin, Kaempferol, and Quercetin Powders and Solutions

**DOI:** 10.3390/ijms25052558

**Published:** 2024-02-22

**Authors:** Alexandra Deriabina, Tatiana Prutskij, Hector Daniel Morales Ochoa, Eduardo Gonzalez Jimenez, Sergei Deriabin

**Affiliations:** 1 Faculty of Physical and Mathematical Sciences, Autonomous University of Puebla (BUAP), Puebla 72570, Mexico; alexandra.deriabina@correo.buap.mx (A.D.);; 2 Sciences Institute, Autonomous University of Puebla (BUAP), Puebla 72570, Mexico; 3 Institute for Biological Instrumentation, Pushchino 142290, Russia

**Keywords:** myricetin, quercetin, kaempferol, flavonoids, fluorescence, TDDFT, ESIPT, exited state proton transfer

## Abstract

Myricetin is a flavonol with high antioxidant properties. In this research, the fluorescence emission of myricetin powder and its solutions in different solvents were measured and analyzed by comparing with the results of calculations. Comparison of the calculated and measured characteristic wavelengths allowed the identification of all the spectral features in the fluorescence spectra of myricetin powder and solutions with different concentrations. The computation was based on modeling the process of the excited state intermolecular proton transfer, which predicts the formation of tautomeric forms of the flavonol molecule. Characteristic emission wavelengths were obtained using TDDFT/M06-2X/6-31++G(d,p). To understand the influence of the hydroxyl groups in the B-ring of the flavonol molecule on the emission spectrum, we also compared the fluorescence spectra of myricetin with those of kaempferol and quercetin. Moreover, based on the analysis of the changes in the shape of the FL spectra with the concentration of the solution, a criterion for the complete dissolution of the flavonol powders was established, which is important for bioavailability of flavonoids.

## 1. Introduction

Flavonoids are important components of human diet, found in fruits, vegetables, coffee, tea, and infusions. There is a growing interest in their therapeutic potential due to their many beneficial properties, including anti-inflammatory, antioxidant, antitumor, and cardioprotective effects [1,2,3,4]. In addition, flavonoid’s molecules are known to act as metal chelators; they play an important role in increasing the bioavailability of metal ions, such as Al, in the human body and can reduce toxicity of heavy metals such as Pb [3]. Antiviral activity of some widely distributed flavonoids such as quercetin (Q), kaempferol (KMP), and myricetin (MYR) were reported in [3]; moreover, these compounds are now considered as important candidates for the treatment of SARS-CoV2 [5,6,7,8,9]. Furthermore, MYR was also found to reverse the effects of high-fat diet on body weight gain, as well as on visceral fat pad weights [10]; it has been shown to act as an antidiabetic agent [1,3] and as an antialcoholic agent [11]. Several studies have analyzed the possibility of increasing the solubility of flavonoids powders to improve their bioavailability [12,13].

**Figure 1:**
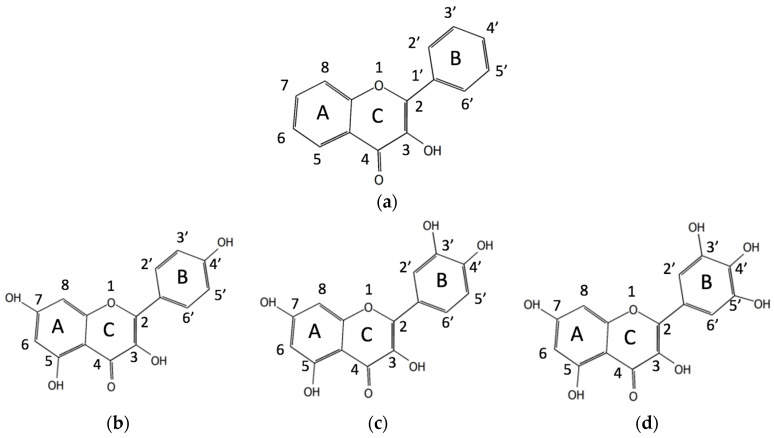
(**a**) Flavonol backbone; (**b**) molecular structure of KMP; (**c**) molecular structure of Q; (**d**) molecular structure of MYR.

Q, KMP, and MYR belong to the subgroup of flavonoids named flavonols that have a flavone backbone with a carbonyl group in the fourth position and a hydroxyl group (OH group) in the third position, as is shown in Figure 1a. The structures of these three molecules differ in the number of hydroxyl groups in the B-ring: KMP has an OH group in the 4′ position (Figure 1b), Q—in the 4′ and 3′ positions (Figure 1c), and MYR has OH groups in positions 3′, 4′, and 5′ (Figure 1d).

The difference in the number of OH groups in the B-ring of KMP, Q, and MYR molecules influences some of their properties. For example, it was recently shown [14] that due to the greater number of hydroxyl groups in the MYR molecule, it has stronger activity in free radicals scavenging. Another property for which the number of OH groups is important is the antioxidant capacity of flavonoids, attributed to their ability to protect cells from oxidative stress and to decrease the risk of cardiovascular diseases, cancer, and neurodegenerative disorders [1,2]. Several studies have shown that MYR is a stronger antioxidant than Q and KMP: in [15], the order of antioxidant activity in brain neurons was found to be MYR > Q > KMP, and in [16], it was shown that the order of their activity in liposomes is MYR > Q.

In the last decade, some studies have been carried out on the optical properties of MYR. In the absorption spectrum of MYR solution in DMSO-phosphate buffer, two bands at 253 nm and at 370 nm were observed [14]. In [17], changes in absorption spectra due to variation of pH in the Britton–Robinson buffer with the concentration of 10 μM were studied. In an acidic environment, the spectra showed the same peaks as those shown in [14], and increasing pH resulted in a red shift for both peaks. In [18], it was shown that the maximum of the absorption spectra of MYR solutions in different solvents, with the concentration of 5 µM, was in the range of 350–400 nm and depended slightly on the type of solvent. In that study, the fluorescence (FL) emission spectra of solutions with the same low concentration (5 μM) of galangin, Q, KMP, and MYR were also compared. The FL spectra of all their solutions in different solvents had the FL peak with the maximum in the range of approximately 490–520 nm. In addition, MYR was shown to have the lowest FL intensity among those flavonols.

Most of the flavonols display dual FL, which is attributed to photoinduced excited state intramolecular proton transfer (ESIPT) [18,19,20,21,22] between the hydroxyl (OH) group and an adjacent carbonyl group in the A–C rings (Figure 1a). Q, KMP, and MYR molecules have –OH3 and –OH5 hydroxyl groups (see Figure 1b–d) that can act as H-bond donors for the carbonyl O4 atom; therefore, these molecules can have keto O3 and keto O5 tautomeric forms after the transfer of proton H3 (H5) to O4 atom.

Earlier, we studied the emission properties of flavonoids Q [21], morin [22], and KMP [23]. Here, we extended that study to another flavonoid, MYR. The main purpose of this research was to understand the influence of the hydroxyl group on the FL emission properties by comparing the FL spectra obtained on flavonols with similar molecular structures, except for B-ring hydroxylation: MYR, KMP, and Q. In addition, the dependence of the FL spectra on the MYR concentration in solutions was analyzed, and the FL spectra of MYR solutions were compared with those of the MYR powder. Further, the calculated characteristic wavelengths of the MYR molecule absorption and emission were compared with those obtained from FL spectra. To the best of our knowledge, neither study of MYR solutions with high concentration nor of the FL emission of MYR powder have yet been reported.

## 2. Results and Discussion

### 2.1. Characteristic Wavelengths of Absorption and Emission of MYR Molecule and Its Ions Obtained at TDDFT/M06-2X/6-31++G(d,p) Level of Theory 

**Figure 2:**
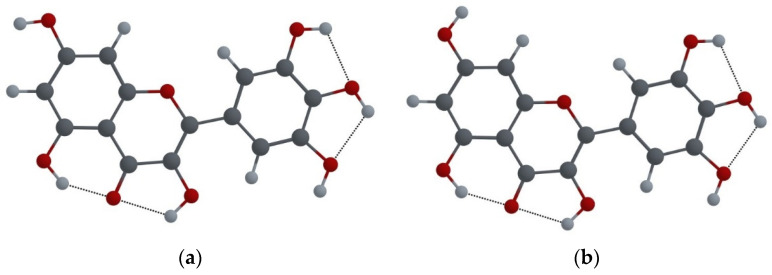
(**a**) Conf.I of MYR molecule; (**b**) Conf.II of MYR molecule.

The MYR molecule has six hydroxyl groups (see Figure 1d), three of which (at the positions 3, 5, and 7) can rotate 180°; therefore, due to their different mutual orientation, 23 different configurations of the molecule are possible. For three hydroxyl groups in the ring B (at the positions 3′, 4′, and 5′), the number of mutual orientations is limited since two hydrogen atoms of neighboring groups cannot be directed towards each other, so only configurations having OH groups in the same plane are possible, thus making a total of 32 different probable configurations of the MYR molecule. For all of them, we performed geometry optimization using the DFT/M06-2X/6-31++G(d,p) method. The resulting lowest energy configuration (Conf. I) is shown in Figure 2a. Shown in Figure 2b is also the configuration found in a crystal of MYR hydrate (Conf. II) [13,24]. The only difference between these two configurations is the orientation of the –OH7 group, and the corresponding difference in their energies is very small, less than 0.3 kcal/mol.

**Figure 3:**
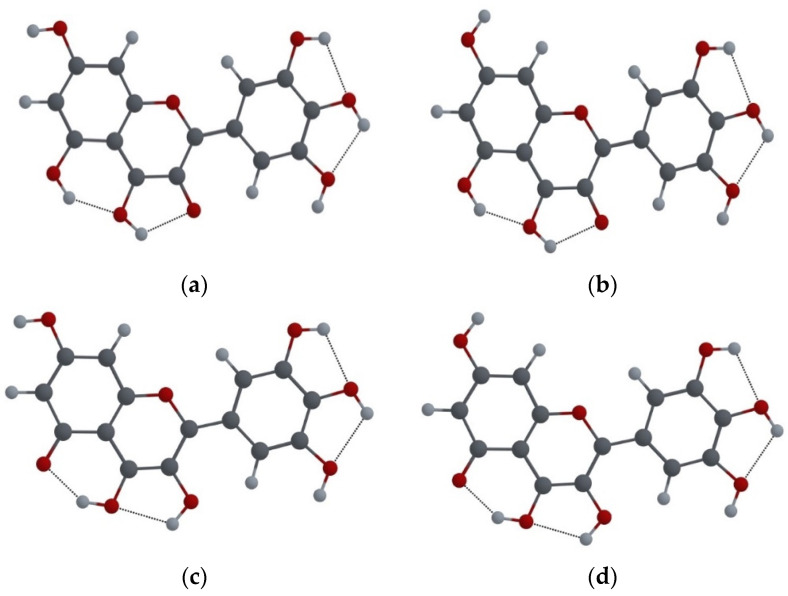
(**a**) Keto O3 form of Conf.I of MYR molecule; (**b**) keto O3 form of Conf.II of MYR molecule; (**c**) keto O5 form of Conf.I of MYR molecule; (**d**) keto O5 form of Conf.II of MYR molecule.

It was already mentioned that to describe the FL emission of a flavonoid molecule, the ESIPT process model is commonly used. The MYR molecule can have two keto forms in the excited state, which arise as a result of the transfer of proton from the –OH3 (–OH5) group to the O4 carbonyl atom. Shown in Figure 3 are these keto forms for Conf.I and Conf.II.

**Figure 4:**
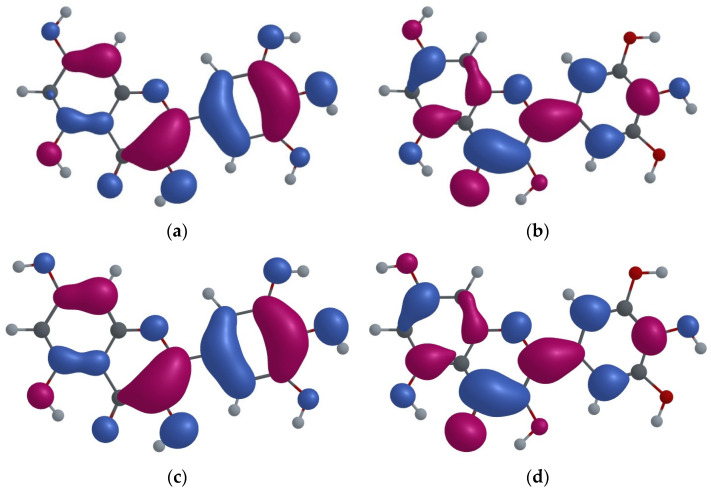
(**a**) Electron population of the HOMO of Conf.I of MYR molecule; (**b**) electron population of the LUMO of Conf.I of MYR molecule; (**c**) electron population of the HOMO of Conf.II of MYR molecule; (**d**) electron population of the LUMO of Conf.II of MYR molecule.

The knowledge of the shape of electron population of a molecule’s orbitals provides information about its electronic structure and the bonding between its atoms [25]; therefore, frontier molecular orbitals (FMO) analysis for both MYR configurations was also performed. The highest occupied molecular orbital (HOMO) indicates the regions of the molecule where electron density is concentrated, while the lowest unoccupied molecular orbital (LUMO) represents the regions to where electrons can be excited when light is absorbed. The HOMO and LUMO for Conf.I and Conf.II are shown in Figure 4.

Figure 4 shows that the difference between Conf.I and Conf.II does not significantly affect the electronic density of HOMO and LUMO orbitals. The HOMO of both enol conformations shows a predominant π-bonding character, while the LUMO shows a general π* antibonding character. Furthermore, the electronic density of oxygen O4 in both configurations increases within the LUMO (with respect to HOMO), while the electronic densities of O3 and O5 decrease. Consequently, in the first excited state, the oxygen atom O4 more strongly attracts the protons associated with the oxygen atoms O5 and O3, which provides stability to the keto O5 and keto O3 forms, making them stable in the excited state.

**Figure 5:**
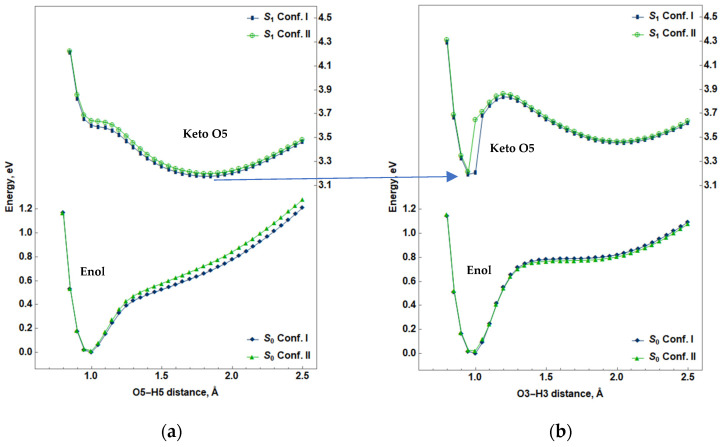
(**a**) Dependence of the potential energy of the MYR molecule on the distance between O5 and H5 atoms in the ground state (S_0_) and in the first excited state (S_1_) for Conf. I and Conf. II; (**b**) dependence of the potential energy of the MYR molecule on the distance between O3 and H3 atoms in the ground state (S_0_) and in the first excited state (S_1_) for Conf. I and Conf. II.

To better understand the ESIPT process in the MYR molecule, the relaxed scan of the potential energy of the MYR molecule as a function of the distance between oxygen and hydrogen atoms for –OH3 and –OH5 hydroxyl groups was performed; i.e., for each fixed distance between the O5 and H5 atoms (O3 and H3) in the range 0.8–2.5 Å, the potential energy minimum of the MYR molecule with respect to the values of all other internal coordinates of the molecules was obtained. The resulting curves for Conf.I and Conf.II in the ground state and in the first excited state are shown in Figure 5.

Our calculations show that the energy dependence on the O3–H3 and O5–H5 distances for Conf. I are very similar to those of Conf. II: for the ground state, both configurations in the enol form have a minimum corresponding to the O3–H3 distance of 0.975 Å, and to the O5–H5 distance of 0.983 Å. Also, it can be seen that there are no minima in the potential energy curves corresponding to the keto O5 and keto O3 forms in the ground state.

The energy dependence for Conf.I and Conf.II on the distance between O5 and H5 atoms (Figure 5a) for the first excited state S_1_ has the saddle point for the enol form and a broad minimum for the keto O5 form, corresponding to the O5–H5 distance of 1.85 Å and the O4–H5 distance of 0.98 Å. This curve demonstrates the viability of the spontaneous ESIPT process for the H5 atom.

The energy dependence on the O3–H3 distance for the first excited state S_1_ for Conf. I and Conf. II, shown in Figure 5b, has two minima. The first minimum at the O3–H3 distance of 0.95 Å does not correspond to the enol form, but to the keto O5 form, for which the potential energy is the lowest. This is consistent with the potential energy curve shown in Figure 5a, which indicates that the keto O5 form corresponds to the lower energy of the first excited state. As the O3–H3 distance increases and approaches 2.0 A, the keto O3 form becomes stable. The difference between the minimum energies of the keto O5 and keto O3 forms is approximately 0.26 eV, and the value of the energy barrier between them is 0.38 eV.

Thus, the analysis of FMO electron population shows a preference for keto forms (relative to the enol form) in the excited state, whereas the relaxed scan of the MYR molecule in the first excited state shows clear preference for the keto O5 form (not for the keto O3 form). 

Using the time-dependent density functional theory (TDDFT) [26,27,28] approach together with the Minnesota functional M06-2X [29] and 6-31++G(d,p) basis set, absorption and emission wavelengths (λ_ab_ and λ_em_) for enol and two keto forms of the MYR molecule were calculated. The results are shown in Table 1.

Taking into account the dependence of the MYR molecule energy on the distances between the H5 proton (H3) and the O5 (O3) atom, presented in Figure 5, which shows that only the enol form is stable in the ground state, Table 1 displays only this form for the ground state of Conf.I and Conf.II. Both keto forms have minima in the first excited state, but not in the ground state; thus, the FL emission corresponding to radiative transitions from the energy minima is presented for the keto forms only.

Calculated wavelength of absorption for enol forms of Conf. I and Conf. II is approximately 320 nm. The emission wavelengths for the keto O3 (O5) forms are 497 nm (625 nm) for Conf. I and 493 nm (604 nm) for Conf. II.

The energy of the keto O5 in the first excited state is lower, at approximately 6 kcal/mol, than that of the keto O3 for Conf.I and Conf.II. Also, according to Figure 5a during optimization of the enol form in the first excited state, the transfer of the H5 proton towards O4 atom occurs, leading to the formation of the keto O5; therefore, the emission of the keto O5 form of the MYR molecule is more probable than that of the keto O3.

In the crystal structure of the MYR hydrate shown in [13], several H-bonds between neighboring MYR molecules can be observed. Through these intermolecular H-bonds, the excited state proton transfer (ESPT) between two neighboring molecules can take place, leading to the formation of positively and negatively charged ions of the MYR molecule. Characteristic absorption and emission wavelengths of those possible ions for Conf.II are also shown in Table 1. The crystal structure of the MYR hydrate allows the formation of two cations with an additional proton in the 3′ and 4′ positions, and, due to the absence of the proton at the 3, 7, 3′ or 5′ positions, the formation of four different anions is also possible.

Our computations show that spontaneous ESIPT of the H5 proton, such as in the neutral molecule, is also possible within some of these ions; for example, the cation 3′ has only enol form in the ground state and keto O5 form in the first excited state. For most of those ions (except for the cation 3′), the enol forms are stable in the first excited state.

For all possible ions of the MYR molecule, the energy minimum in the first excited state corresponds to the enol form of the 3’ anion, with the calculated characteristic wavelength of absorption (emission) of 509 nm (639 nm). Our calculations also yield values for an absorption (emission) wavelength of 566 (686) nm for the anion corresponding to the absence of the proton at the 5’ position in the B-ring. The keto O3 form of 3′ and 5′ anions have the emission wavelengths of 904 nm and 944 nm, respectively. Keto O5 forms of these anions are not stable either in the ground or in the excited state. Thus, both anions in the B-ring show a similar behavior with a slight difference up to 60 nm in absorption and emission wavelengths.

### 2.2. FL Spectra of MYR, KMP, and Q Hydrate Powders

**Figure 6:**
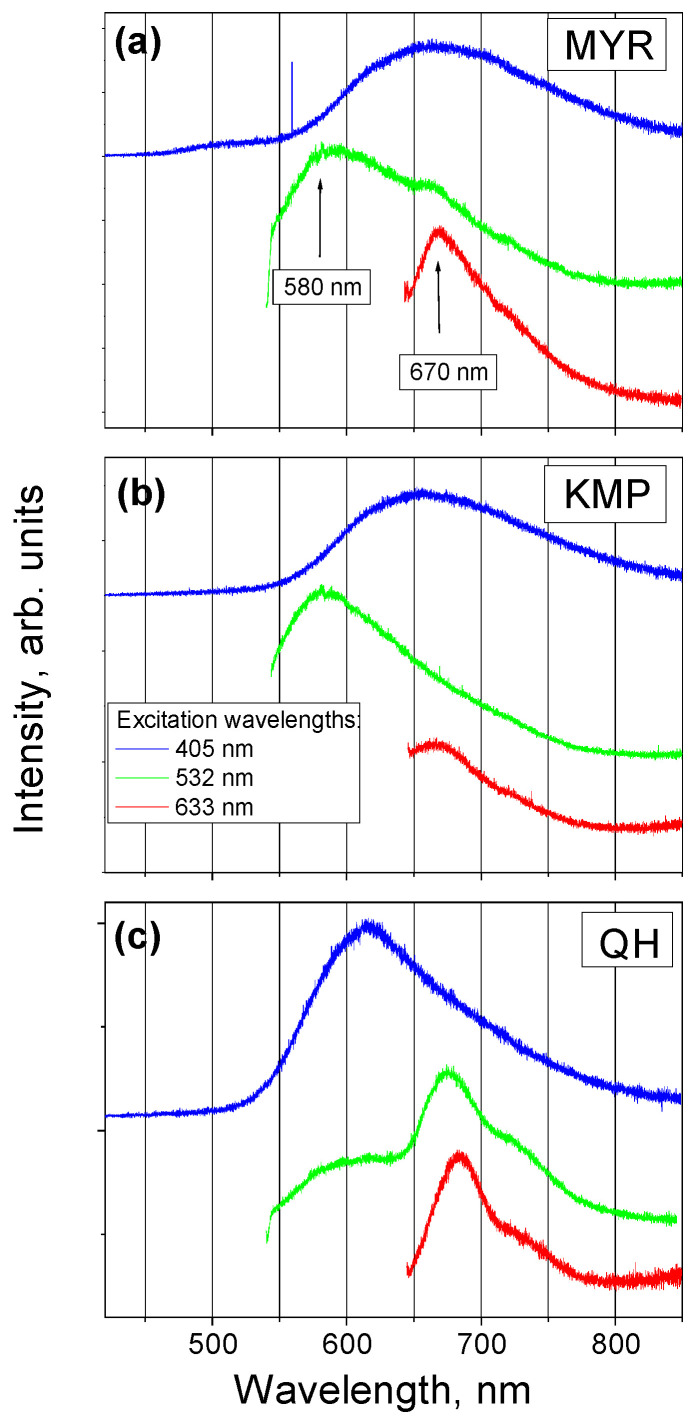
(**a**) FL spectra of the MYR powder; (**b**) FL spectra of the KMP powder; (**c**) FL spectra of the QH powder. The spectra were obtained using the excitation wavelengths of 405 nm (blue lines), 532 nm (green lines), and 633 nm (red lines). For the sake of clarity, all the spectra were normalized to unity at the maximum value, set on the same scale and shifted vertically.

To reveal different levels of emission energy of the MYR molecule, we measured the FL spectra using three different excitation wavelengths: 405, 532, and 633 nm. The FL spectra of the MYR, KMP, and Q hydrate (QH) powders are shown in Figure 6.

To identify the FL emission bands, we compare the spectra of the MYR, KMP, and QH powders. The FL spectra of the MYR and KMP powders obtained using the highest excitation energy, corresponding to the lower wavelength of 405 nm, are very similar: the spectra are broad, with the maximum at approximately 650 nm. The FL spectrum of QH is also broad, with the maximum at 620 nm. KMP and MYR powders also have very similar FL spectra under lower excitation energy, corresponding to 532 nm; the maxima of those spectra are located at 580 nm. An additional spectral feature at 670 nm is observed in the spectrum of MYR powder. The lowest excitation energy, corresponding to the wavelength of 633 nm, reveals that spectral feature more clearly. The same feature at 670 nm, but with lower intensity, is also observed in the spectrum of KMP powder obtained with the lowest excitation energy. The FL emission of QH powder under lower excitation, corresponding to 532 nm (633 nm), shows two (one) FL bands at 580 nm and 670 nm (at 670 nm); i.e., it contains all the peaks found in the spectra of KMP and MYR powders, but their intensities are different. 

The FL peak at 670 nm in the spectrum of the MYR powder is consistent with the calculated value of 686 nm corresponding to the emission wavelength of the 5′ anion of the MYR molecule. The corresponding calculated absorption wavelength for that ion is 566 nm; therefore, considering the approximation of our model, the emission band at 670 nm can be excited by the energies corresponding to both wavelengths: 532 nm and 633 nm. On the other hand, calculations show that the FL emission wavelength corresponding to the 3’ anion of the MYR molecule is 639 nm; therefore, it can also contribute to that FL peak.

The FL peak at 580 nm is due to emission of the keto O5 form of the MYR molecule. In our previous work [23], we showed that the emission of keto O5 forms of KMP and Q molecules are very close to this value. The broad spectral feature at 650 nm observed under excitation wavelength of 405 nm can contain the contribution of the emission of the keto form O5 of the molecule together with that of all the ions. 

### 2.3. Calculated Characteristics Wavelengths of Absorption and Emission of MYR Molecule in Different Solvents 

In Table 2, we show calculated characteristic wavelengths of absorption and emission of different tautomeric forms of Conf. II of the MYR molecule in methanol and DMSO.

As can be seen from Table 2, the results obtained for Conf.II are almost the same. The absorption and emission wavelengths for both solvents are also very close. The presence of solvent makes the keto O3 form stable in the ground state, and the corresponding absorption wavelength value is 406 nm. The energies of the first excited state for the two keto forms are very close to each other, while the energy corresponding to the keto O5 form is lower than for the keto O3 form. The calculated value for the emission wavelength for the keto O5 form is 561 nm for the solution in methanol and 557 nm for the solution in DMSO. For emission wavelength corresponding to the keto O3, our calculations display the value of 483 nm in methanol and of 473 nm in DMSO. The oscillator strength for the keto O3 form emission is higher than that obtained for the keto O5 form.

### 2.4. FL Spectra of MYR and KMP Solutions

**Figure 7:**
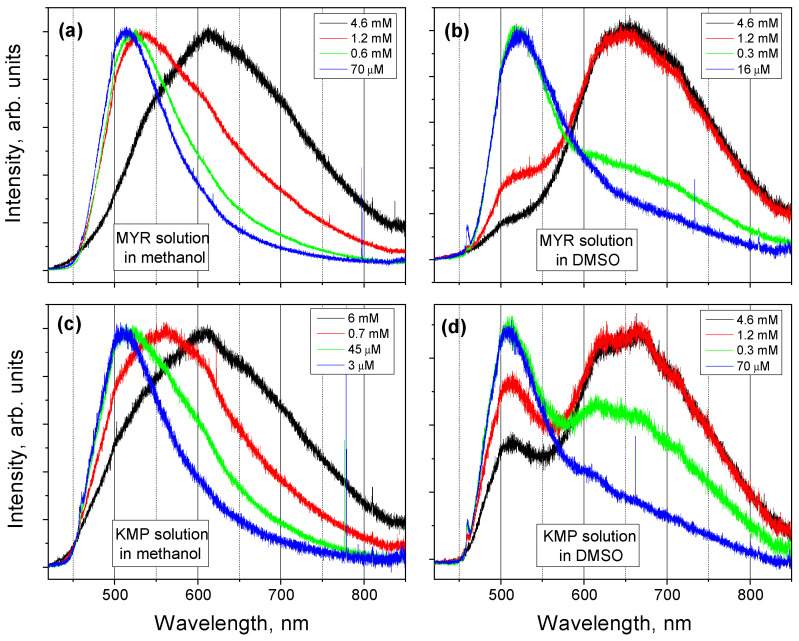
(**a**) FL spectra of MYR solutions with different concentrations in methanol; (**b**) FL spectra of MYR solutions with different concentrations in DMSO; (**c**) FL spectra of KMP solutions with different concentrations in methanol; (**d**) FL spectra of KMP solutions with different concentrations in DMSO. All the spectra are normalized to unity at its maximum value.

KMP and MYR are soluble in different types of solvents, as are many of flavonoid powders. The solubility of KMP and MYR powders in polar solvents is significantly higher than in nonpolar ones. We chose methanol, a polar protic solvent, since many flavonoids have the highest solubility in that solvent, and DMSO, a polar aprotic solvent, because it is often used in medicine as a solvent for various drugs and is also used in cosmetic and pharmaceutical industries. Shown in Figure 7 are the FL spectra of KMP and MYR solutions in those solvents.

The spectra of MYR and KMP solutions are very similar: the spectra of the solutions in methanol (DMSO) with higher concentration contain two spectral features at 510 nm and at 620 (650) nm. Comparison with the calculation results allows the identification of the FL peak at 510 nm as related to the emission of both keto forms (O3 and O5) in solution (see Table 2), and of the peak at 620 nm as related to the emission of the keto form of O5 of the MYR molecule (see Table 1). When the solution concentration decreases, the contribution of the feature at higher wavelength decreases, and the spectra of the solutions with lowest concentration contain only the peak at the lower wavelength. This can be explained as follows: in solutions with high concentration, undissolved clusters of solute molecules remain, and, therefore, the maximum of the FL spectrum of those solutions is located at the same wavelength as that of the FL spectrum of powder. When all the solute molecules are separated from each other, the FL spectrum of solution contains only the peak corresponding to the molecule surrounded by solvent molecules.

Based on this observation, a criterion for the complete dissolution can be established: when only one peak at the shorter wavelength is observed in the FL spectrum of solution, all the solute molecules are separated from each other. In this way, the solubility of the flavonoid powder can be assessed and compared with the solubility of other powders.

**Figure 8:**
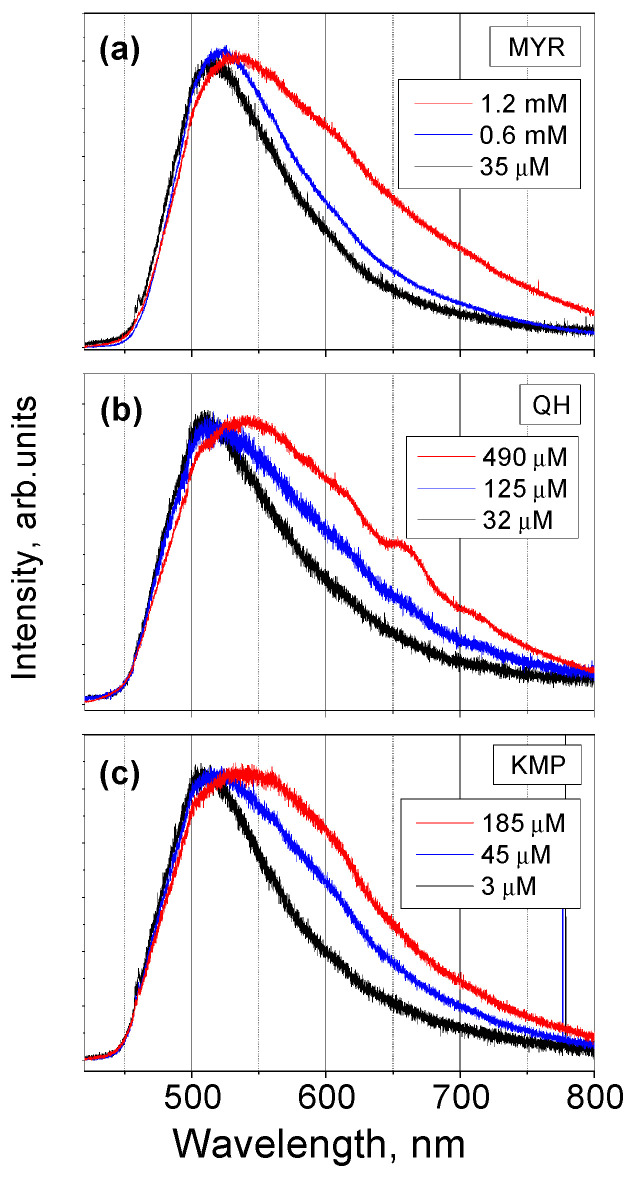
(**a**) FL spectra of MYR solutions in methanol with different concentrations; (**b**) FL spectra of QH solutions in methanol with different concentrations; (**c**) FL spectra of KMP solutions in methanol with different concentrations. All the spectra are normalized to unity at its maximum value.

An exact numerical criterion for the concentration needed for the solute molecules’ separation cannot be set, since the process of dissolution is gradual and the solution FL spectrum changes little by little; however, a relative criterion can be established by comparing the shape of the spectra. In Figure 8, the FL spectra of MYR, QH, and KMP solutions with different concentrations are shown. The spectra corresponding to solution concentrations of 600 µM (MYR), 32 µM (QH), and 3 µM (KMP) have almost the same shape. With a further decrease in concentration, the shape and the spectral position of the FL maximum do not change. The shapes of the spectra of solutions with higher concentrations of 1.2 mM (MYR), 490 µM (QH), and 125 µM (KMP) also look similar, and are slightly shifted towards longer wavelengths.

For different flavonoid powders, the concentrations corresponding to the solutions without clusters are different, and a tendency can be observed: the solubility positively correlates with the number of hydroxyl groups in the flavonoid’s molecule, i.e., the more hydroxyl groups in a molecule, the higher the solubility of its powder in methanol. It can be explained as follows: the increased number of hydroxyl groups in the flavonoid’s molecule increases the number of possible hydrogen bonds with methanol molecules, thus improving the solubility of the powder. 

This finding is consistent with the results reported in [30], where a new efficient and environmentally friendly extraction method using the water as extractant (subcritical water extraction) was studied to obtain KMP, Q, and MYR from natural ingredients such as black tea, celery, and ginseng leaf. The maximum yield for KMP was at higher temperature than for MYR and Q, since KMP has a smaller number of hydroxyl groups and, therefore, the lowest solubility. The observed dependence of the solubility on the number of hydroxyl group in the solute molecule can also explain the results of [15], in which the antioxidant activity in brain neurons of MYR, Q, and KMP were analyzed. It was found that the activity of MYR is higher than that of Q, which in turn is higher than that of KMP. 

In summary, we studied FL properties of MYR powder and its solutions in methanol and DMSO with different concentrations. In order to understand the effect of the hydroxyl groups on the emission properties of the flavonol molecule, we compared the measured FL spectra of MYR, KMP, and Q, since KMP and Q molecules have molecular structure similar to that of MYR, except for the hydroxylation of the B-ring. 

We calculated the characteristic absorption and emission wavelengths for the MYR molecule using the TDDFT/M06-2X/6-31++G(d,p) approach and for the MYR molecule in solution using the PCM/ TDDFT/M06-2X/6-31++G(d,p) method and compared these values with those obtained from the measured FL spectra. 

We performed frontier molecular orbital population analysis, which showed the preference for the keto form, with respect to the enol form, in the excited state, which leads to the excited state intramolecular proton transfer of the H3 (H5) proton to the O4 atom. In addition, the relaxed scan performed as the function of the O3–H3 and O5–H5 distances showed the preference for the keto O5 form over the keto O3 form.

We identified the spectral features of the experimentally obtained FL spectra of MYR powder and MYR solutions by comparing their wavelengths with calculated values. Our calculations considered the ESIPT process within the MYR molecule and the exited state proton transfer (ESPT) to the neighboring MYR molecule in the crystal. According to this analysis, the characteristic features of the spectra correspond to the following: in the FL spectrum of the MYR powder, the broad band with the maximum at 650 nm contains the contribution of the radiative transition corresponding to the keto O5 and also the contribution of the emission of several ions.

The usage of lower excitation energies revealed two additional features at 670 nm and 580 nm. The first of them, at 670 nm, corresponded to the emission of the enol form of 5′ anion or/and to the enol form of the 3′ anion of the MYR molecule. The second one, at 580 nm, was due to the radiative transition of the keto O5 of the MYR molecule.

We analyzed the dependence of the FL spectra on the concentration of MYR in solutions and compared their FL spectra with the FL spectra of MYR powder. The FL spectra of solutions with high concentrations showed the same feature as that observed in the spectrum of the powder, pointing to the presence of clusters of molecules. If the concentration decreases, the FL peak maximum shifts towards lower wavelengths. The FL spectra of solutions with low concentration showed only one peak with the maximum at 510 nm, which can be due to emission of both keto O3 and keto O5, forms of the MYR molecule.

Based on the analysis of the dependence of the FL spectra of MYR, Q, and KMP solutions on concentration, it is possible to establish a criterion for complete dissolution of the powder: all molecules of the solute are separated from each other if, with decreasing concentration, the FL spectrum of the solution, shifted towards shorter wavelengths, does not change its shape, but only its intensity. 

Our study showed that FL properties of flavonol molecules depend little on the number of OH groups in the B-ring. The FL spectra of powder and solutions of MYR and KMP, i.e., compounds with the smallest and largest number of hydroxyl groups in the B-ring, are very similar, which confirms that the process determining the FL emission spectrum, ESIPT, is independent of the B-ring, since it occurs in a different part of the molecule. In contrast, the solubility of these flavonol powders varies and largely depends on the OH groups number.

## 3. Materials and Methods

### 3.1. Materials

Myricetin (M6760 powder with >96% purity), kaempferol (60010 powder with >97% purity), and quercetin hydrate (337951 powder with >95% purity) were acquired from Sigma-Aldrich. Methanol (>99.5%) and dimethyl sulfoxide (DMSO) (>99.9%) were purchased from J. T. Baker. 

### 3.2. Experimental Setup

The FL emission was measured using a conventional experimental setup, which included a TRIAX550 monochromator (Horiba/Jobin Yvon) and a liquid-nitrogen-cooled charge-coupled-device detector (Princeton Instruments). The optical excitation was provided by a laser diode with a wavelength of 405 nm, a solid-state laser with 532 nm, or a helium-neon laser with 633 nm. To avoid degradation, a low-excitation light intensity of approximately 5 mW was used in all measurements.

### 3.3. Computation Details

For full geometry optimization and for vibrational frequency calculations, the density functional theory (DFT) [26,27] approach was used with the Minnesota functional M06-2X [29] and 6-31++G(d,p) basis set, implemented in Gaussian 16 [31]. We chose this functional since it reproduces well the nonplanarity of flavonoid molecules in the ground state, which is important for accurately assessing the characteristic wavelengths of the flavonoids FL emission [32]. For the excited states computation, the time-dependent DFT (TDDFT) [28] approach was used. The influence of the solvents on the FL emission energy was simulated by using the polarizable continuum model (PCM) within the self-consistent reaction field (SCRF) method [33]. All the calculations were performed using the supercomputer facility of Laboratorio Nacional de Supercomputo del Sureste (LNS), Mexico.

## 4. Conclusions

Calculations based on the ESIPT and ESPT models made it possible to identify all spectral features of the experimentally obtained FL spectra of MYR powder and MYR solutions. The FL properties of the flavonol molecules were shown to depend little on the number of OH groups in the B-ring, while the solubility of MYR, Q, and KMP powders changed strongly with the number of OH groups. It was possible to establish a criterion for complete dissolution of these powders.

## Figures and Tables

**Table 1:** Absorption and emission wavelengths for different keto forms of MYR molecule and MYR ions obtained at TDDFT/M06-2X/6-31++G(d,p) level of theory.

	E_S0_ (a.u.) S_0_ opt	E_S1_(a.u.) S_0_ opt	E_S1_ − E_S0_ (eV)	λ_ab_ (nm)	f_ab_	E_S1_* (a.u.) S_1_ opt	E_S0_* (a.u.) S_1_ opt	E_S1_* − E_S0_* (eV)	λ_em_ (nm)	f_em_
Conf.I										
Enol	−1179.0360	−1178.8949	3.8697	320.90	0.57	-	-	-	**-**	-
Keto O3	-	-	-	-	-	−1178.9091	−1179.0006	2.49	497.53	0.50
Keto O5	-	-	-	-	-	−1178.9191	−1178.9945	2.05	604.52	0.09
Conf.II										
Enol	−1179.0356	−1178.8934	3.8692	320.44	0.57	-	-	-	-	-
Keto O3	-	-	-	-	-	−1178.9085	−1179.0008	2.509	493.99	0.49
Keto O5	-	-	-	-	-	−1178.9182	−1178.9911	1.982	625.43	0.07
Ions of Conf.II										
Anion 3 enol	−1178.4926	−1178.3825	2.9961	413.82	0.0001	−1178.3912	−1178.4837	2.5173	492.54	0.0001
Anion 3 keto O5	-	-	-	-	-	−1178.3902	−1178.4797	2.4344	509.29	0.43
Anion 3′ enol	−1178.5015	−1178.4121	2.4355	509.07	0.09	−1178.4210	−1178.4923	1.9404	638.97	0.04
Anion 3′ keto O3	-	-	-	-	-	−1178.4061	−1178.4565	1.3714	904.06	0.03
Anion 5′ enol	−1178.4809	−1178.4004	2.1889	566.42	0.11	−1178.4072	−1178.4736	1.8067	686.23	0.07
Anion 5′ keto O3	−1178.4508	−1178.3835	1.8315	676.96	0.12	−1178.3928	−1178.4410	1.3134	944.02	0.04
Anion 7 enol	−1178.5051	−1178.3880	3.1865	389.09	0.35	−1178.4025	−1178.4890	2.3550	526.46	0.20
Anion 7 keto O3	−1178.4892	−1178.3809	2.9488	420.45	0.48	−1178.3949	−1178.4797	2.301	537.18	0.40
Anion 7 keto O5	-	-	-	-	-	−1178.3917	−1178.4558	1.7443	710.78	0.001
Cation 3′ enol	−1179.3312	−1179.1965	3.6651	338.28	0.26	-	-	-	-	-
Cation 3′ keto O3	−1179.3089	−1179.2055	2.8165	440.20	0.44	−1179.2135	−1179.3022	2.4131	513.80	0.41
Cation 3′ keto O5	-	-	-	-	-	−1179.2252	−1179.2789	1.4638	846.98	0.03
Cation 4′ plane	−1179.3966	−1179.2834	3.0823	402.24	0.78	−1179.2867	−1179.3932	2.8995	427.60	0.76

Note: E_S0_ is the energy of the molecule optimized in the ground state S_0_; E_S1_* is the energy of the first excited state, S_1_, at its optimized geometry; E_S0_* is the energy of the ground state, S_0_, at the optimized geometry of the excited state, S_1_; E_S1_ is the energy of the first excited state, S_1_, at the ground state optimized geometry, S_0_. f_em_ and f_ab_ are the oscillator strengths for emission and absorption, respectively.

**Table 2:** Absorption and emission wavelengths for keto forms of Conf.II of the MYR molecule in solvents obtained at TDDFT-M06-2X/6-31++G(d,p) level of theory with PCM approach.

	E_S0_ (a.u) S_0_ opt	E_S1_ (a.u) S_0_ opt	E_S1_ − E_S0_ (eV)	λ_ab_ (nm)	f_ab_	E_S1_* (a.u) S_1_ opt	E_S0_* (a.u) S_1_ opt	E_S1_* − E_S0_* (eV)	λ_em_ (nm)	f_em_
Methanol										
Keto O3	−1179.0272	−1178.9223	2.854	406.08	0.62	−1178.9256	−1179.0198	2.562	483.83	0.77
Keto O5						−1178.9392	−1179.0204	2.209	561.22	0.41
DMSO										
Keto O3	−1179.0276	−1178.9177	2.9905	414.59	0.63	−1178.9260	−1179.0222	2.6179	473.61	0.7801
Keto O5	-	-	-	-	-	−1178.9395	−1179.0212	2.2237	557.57	0.4274

Note: E_S0_ is the energy of the molecule optimized in the ground state S_0_; E_S1_ is the energy of the first excited state, S_1_, at the ground state optimized geometry, obtained from the nonequilibrium solvation state-specific calculation. E_S1_* is the energy of the first excited state, S_1_, at its optimized geometry from the equilibrium solvation state-specific calculation. E_S0_* is the energy of the ground state, S_0_, with nonequilibrium solvation, at the optimized geometry of the excited state.

## Data Availability

Data are contained within the article and can also be obtained from the corresponding author.
